# O.2.2-8 Delapré Walk Project: are signposted walking routes an effective intervention to increase engagement in urban parks? –Natural experimental study

**DOI:** 10.1093/eurpub/ckad133.123

**Published:** 2023-09-11

**Authors:** Declan Ryan, Jack Hardwicke, Kimberley Hill

**Affiliations:** University of Northampton, UK; declan.ryan@northampton.ac.uk; University of Northampton, UK; declan.ryan@northampton.ac.uk; University of Northampton, UK; declan.ryan@northampton.ac.uk

## Abstract

**Purpose:**

Investigate the effect on visitors' greenspace engagement by retro-fitting wayfinding, to create a looped walking route, within an urban park.

**Methods:**

1. An online-survey was distributed (23^rd^ March – 3^rd^ May 2021) to determine public perceptions of wayfinding's role for recreational physical activity within the urban park and explore what information should be included on wayfinding.

2. Automated and manual counts were monitored on footpaths where the new wayfinding was installed at baseline (4^th^ March – 26^th^ August 2021) and follow-up (29^th^ August 2021 – 29^th^ August 2022). A QR code accessed intercept survey was provided on the wayfinding to understand route-user visitation habits.

3. Intercept go-along interviews were conducted on 8^th^, 14^th^, 24^th^ September 2022 to explore visitor’s reasoning for using the park and engagement with the wayfinding intervention.

**Results:**

Phase 1: Themes from 266 survey respondents suggested wayfinding could increase visitors’ confidence to explore, perceptions of safety, motivation to walk further, and reduced anxiety. Directional arrows, total distance of the route, consistent colours and fonts, a map of the route, local information of interest, and emergency contact information were the main components that respondents strongly agreed should be included on wayfinding.

Phase 2: Manual and automated counts showed no consistent change in daily footfall between baseline and follow-up. However, 23% of route users reported they were following the signs at 12-month follow-up. Intercept survey respondents appeared to be infrequent park users, with the new wayfinding making them feel less anxious about exploring unfamiliar areas, motivating them to walk further than originally planned, and helping them ‘take notice’ of the landscape.

Phase 3: Interviewees (*n*=28) valued the detachment from the urban environment that the park provided, which gave positive experiences to wellbeing, mental health, and social connections. Wayfinding was valued by irregular park users to help them confidently explore the park.

**Conclusions:**

Retro-fitting wayfinding in an urban park helps irregular users engage in the greenspace and wayfinding design needs to integrate with the natural environment to keep contrast with urban environments. The project illustrated the value of qualitative methods within natural experimental studies to capture intervention impacts.

**Support/Funding Source:**

University of Northampton

